# The unfolding COVID-19 pandemic: A probability-based, nationally representative study of mental health in the United States

**DOI:** 10.1126/sciadv.abd5390

**Published:** 2020-10-14

**Authors:** E. Alison Holman, Rebecca R. Thompson, Dana Rose Garfin, Roxane Cohen Silver

**Affiliations:** 1 Sue & Bill Gross School of Nursing, University of California, Irvine, Irvine, CA, USA.; 2 Department of Psychological Science, University of California, Irvine, Irvine, CA, USA.; 3 Departments of Psychological Science and Medicine and Program in Public Health, University of California, Irvine, Irvine, CA, USA.

## Abstract

The COVID-19 (coronavirus disease 2019) pandemic is a collective stressor unfolding over time; yet, rigorous empirical studies addressing its mental health consequences among large probability-based national samples are rare. Between 18 March and 18 April 2020, as illness and death escalated in the United States, we assessed acute stress, depressive symptoms, and direct, community, and media-based exposures to COVID-19 in three consecutive representative samples from the U.S. probability-based nationally representative NORC AmeriSpeak panel across three 10-day periods (total *N* = 6514). Acute stress and depressive symptoms increased significantly over time as COVID-19 deaths increased across the United States. Preexisting mental and physical health diagnoses, daily COVID-19–related media exposure, conflicting COVID-19 information in media, and secondary stressors were all associated with acute stress and depressive symptoms. Results have implications for targeting public health interventions and risk communication efforts to promote community resilience as the pandemic waxes and wanes over time.

## Teaser

As the COVID pandemic unfolded, acute stress and depressive symptoms increased alongside deaths in a U.S. probability-based sample.

**INTRODUCTION**

As the COVID-19 (coronavirus disease 2019) pandemic unfolds across the world, the scientific community has focused on understanding the transmission, biology, and treatment of the novel coronavirus [SARS-CoV-2 (severe acute respiratory syndrome coronavirus 2)]. To date, empirical investigations of the mental health impact of this collective trauma represent less than 3% of the published literature (*1*), even though the pandemic, including its associated social and economic fallout, represents a mental health crisis of unprecedented scope and scale (*2*). Globally, COVID-19 has left hundreds of millions of people at risk for serious illness or death (*3*), isolated in their homes (*4*), and without jobs or income. These circumstances place people living with anxiety, depression, or other mental health challenges at especially high risk for worsening symptoms and suicide (*2*, *5*–*7*).

When faced with ambiguous, ongoing disasters like the COVID-19 pandemic, people often turn to the media for information to guide them (*8*), making media a critical source of exposure to the crisis. Yet, previous research demonstrates that exposure to media coverage of collective traumas, such as mass violence (*9*, *10*), infectious disease outbreaks (*11*), or natural disasters (*12*), may be a double-edged sword that can inform the public while simultaneously amplifying stress symptoms, worry, and perceived risk, with substantive implications for public health (*13*–*15*). Conflicting messages in the media may further exacerbate stress (*16*), especially in the context of coping with life-threatening circumstances that could worsen as the pandemic unfolds over time.

Moreover, the degree to which individuals experience distress as a result of direct exposure to COVID-19 (e.g., contracting the virus) and related secondary stressors (e.g., personal or economic losses, social distancing) varies widely. These different exposures may exacerbate early distress, especially in the context of coping with a collective stressor like the COVID-19 pandemic. For example, analyses of helpline usage data suggest that stricter lockdown orders were associated with more loneliness, anxiety, and suicidal ideation among German helpline users (*17*). However, analysis of Google Trends data suggests that stay-at-home orders may have flattened rising distress as the number of distress-related searches in the United States plateaued soon after the lockdowns began (*18*). At present, little is known about the relative impact of these various exposures—direct, media-driven, or community wide—on individuals’ early pandemic-related psychological responses. Understanding the risk and protective factors affecting public response is critical to promoting community resilience as countries across the globe face a surge of new COVID-19 infections.

From a methodological perspective, the relatively small body of literature addressing COVID-19–related mental health issues has serious flaws that call into question the validity and utility of their findings. For example, only four of the peer-reviewed empirical studies addressing mental health responses to COVID-19 include methodologically rigorous probability-based sampling to enable population inferences (*6*, *19*–*21*), one of which included only young adults (*6*). Rather, the majority of population-based studies have used “snowball” sampling or drawn samples from opt-in, nonrepresentative online panels and then weighted the data to the population—a process that exacerbates the selection biases inherent in opt-in panels and undermines the data’s utility for public policy purposes (*22*). Big data studies (e.g., Google Trends data) also suffer from biases as their samples are self-selected, not probability-based. Last, although one study used a probability-based sample from the U.S. population and documented an increase in psychological distress from 2018 to early postpandemic 2020 (*20*), it did not examine types of exposure, media use, or other predictors of the psychological toll of the pandemic.

**Fig. 1:**
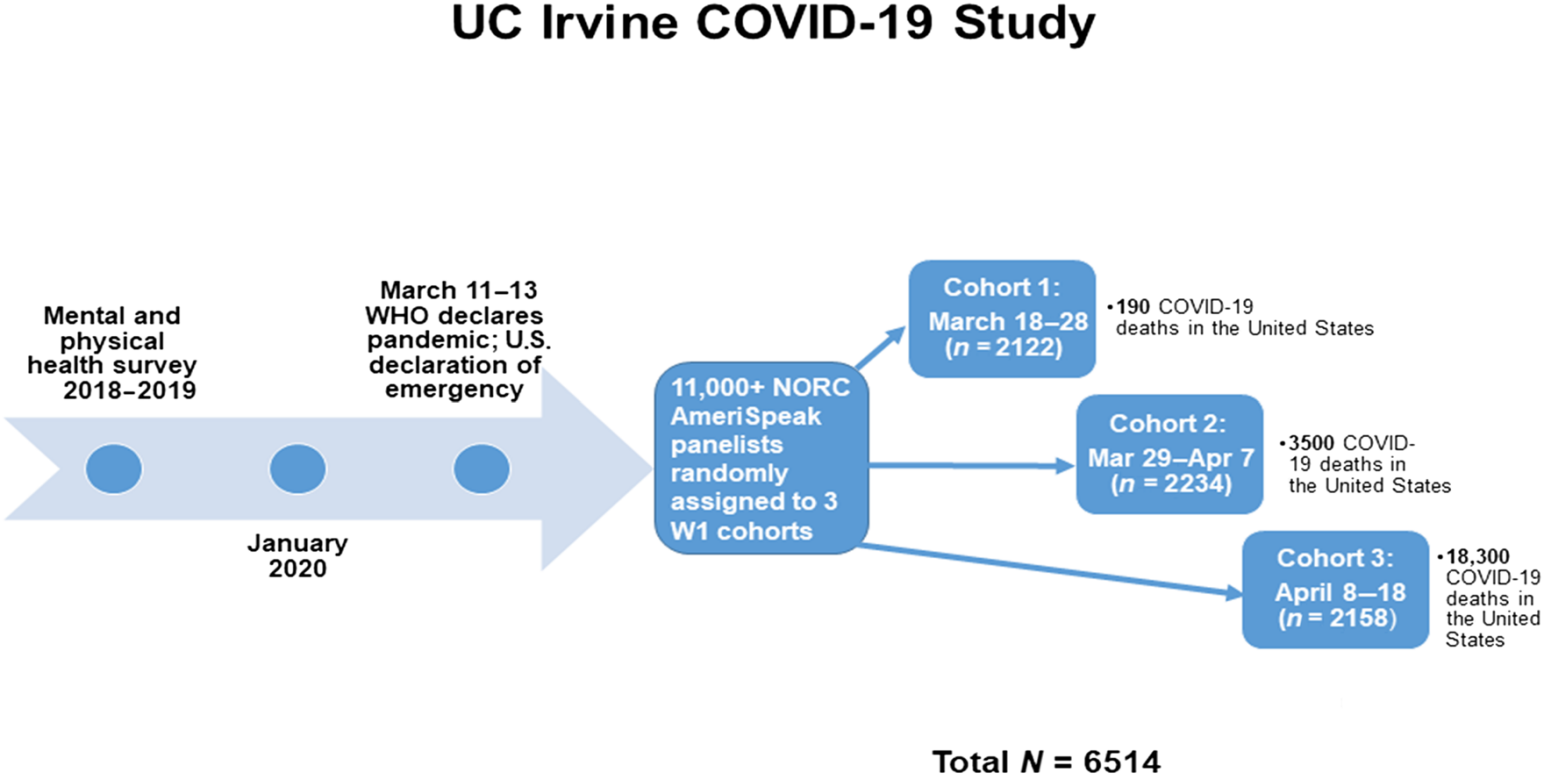
Study design for examining early psychological responses to the COVID-19 pandemic in three consecutive probability-based, nationally representative cohorts in the United States.

Beginning on 18 March 2020 and across the next 30 days, we conducted a rigorous rapid-response study of three consecutive probability-based, nationally representative cohorts in the United States (see Fig. 1) to examine early distress (i.e., acute stress and depressive symptoms) in response to the COVID-19 pandemic. Mental and physical health histories collected before the pandemic provided baseline data and prior research on collective trauma informed appropriate predictors of the outcomes assessed. Over the course of our study, the size of the pandemic shifted dramatically in the United States, from 9415 COVID-19 positive cases and about 190 COVID-related deaths when data collection began for cohort 1, to 124,763 positive cases and about 3500 deaths when data collection began for cohort 2, to 401,166 positive cases and about 18,300 deaths when data collection began for cohort 3 (*3*).

## RESULTS

Three representative cohorts (cohort 1, *n* = 2122; cohort 2, *n* = 2234; cohort 3, *n* = 2158) comprised a final weighted sample (*N* = 6514) that was 51.9% female, ranged in age from 18 to 97 years (*M* = 47.50 years; SD = 17.44), and was 63.6% white (non-Hispanic), 11.8% black (non-Hispanic), 16.0% Hispanic, and 8.7% other ethnicities. One-third of the weighted sample (33.6%) had earned a bachelor’s degree or higher; median annual income was between US$40,000 and US$49,999. Almost two-thirds (66.0%) of the sample lived in an urban area, 10.4% lived in suburbs, 12.9% lived in a town, and 10.6% lived in a rural area. Of the total sample, 17.3% lived in the Northeast region of the United States, 21.0% lived in the Midwest, 37.7% lived in the South, and 24.1% lived in the West. Table S1 provides the weighted sample demographics compared to February 2020 Current Population Survey benchmarks (*23*).

Before the COVID-19 outbreak, participants reported a mean of 1.04 physical health ailments (SD = 1.22), and 17.7% of the sample reported being previously diagnosed with a mental health ailment by a physician. Approximately a quarter of the sample (23.5%) reported that they or a close other had been exposed to COVID-19 (e.g., experienced symptoms, were diagnosed); 29.8% of the sample reported having work-related exposures (e.g., essential/in-person worker). Participants also reported a mean of 4.87 (range: 0 to 6; SD = 1.54) community exposures to the outbreak (e.g., stay-at-home order for their community, school or restaurant closures) and a mean of 1.37 (range: 0 to 7; SD = 1.21) secondary stressors related to the outbreak (e.g., lost job or wages, waiting in long lines for necessary supplies). Media exposure to the outbreak was high; participants reported consuming a mean of 7.06 (range: 0 to 33; SD = 6.91) hours of outbreak-related coverage daily (summed across media sources), consuming more news coverage than pre-outbreak (*M* = 25.99; range: −100 to 100; SD = 47.55), and receiving conflicting information from the news media on average “sometimes” (*M* = 2.95; range: 1 to 5; SD = 1.05).

**Fig. 2:**
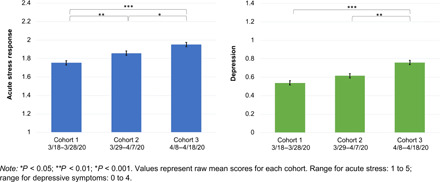
Mean pandemic-related acute stress response and depressive symptoms across cohorts (*N* = 6514). *P < 0.05; **P < 0.01; ***P < 0.001. Values represent raw mean scores for each cohort. Range for acute stress: 1 to 5; range for depressive symptoms: 0 to 4.

Acute stress increased across the three cohorts, with cohort 1 reporting significantly lower acute stress than both cohorts 2 and 3 and cohort 3 reporting significantly higher acute stress than cohort 2 (see Fig. 2). Depressive symptoms also increased over time, with cohort 3 reporting significantly more depressive symptoms than cohort 1 or 2 (see Fig. 2).

Table 1 presents both standardized (β) and unstandardized coefficients for predictors of acute stress and depressive symptoms for the full sample. Prior mental (β = 0.18 and β = 0.27) and physical (β = 0.06 and β = 0.08) health diagnoses were significantly associated with higher acute stress and depressive symptoms, respectively. Demographic characteristics were also important: Females reported higher acute stress (β = 0.12) but not depressive symptoms (β = 0.02), whereas older people (β = −0.10 and β = −0.18) and those who lived in suburban rather than urban areas (β = −0.03 and β = −0.04) reported lower acute stress and depressive symptoms, respectively. Respondents who lived in regions outside of the Northeast (Midwest: β = −0.07, South:β = −0.07, and West:β = −0.06) all reported lower acute stress, but not lower depressive symptoms (Midwest: β = −0.03, South: β = −0.03, and West: β = −0.01) than respondents in the Northeast. Respondents with higher incomes reported lower levels of depressive symptoms (β = −0.08), but not acute stress (β = −0.02).

**Table 1:** Adjusted regression coefficients for OLS regression models predicting pandemic-related acute stress and depressive symptoms to the COVID-19 outbreak (*N* = 6514). Reference group for cohort is cohort 1 (18 to 28 March 2020); reference group for ethnicity is white, non-Hispanic; reference group for residential area is urban; reference group for region is Northeast. All models were estimated using sampling weights to account for sampling design and differences between the sample and U.S. Census benchmarks. Standardized coefficients and confidence intervals were estimated by calculating *z* scores for all model variables (including categorical indicators) and fitting a multiple OLS regression model to the standardized transformation.

	**Acute stress**			**Depressive symptoms**		
**Predictor variables**	**β**	**95% CI**	***b***	**β**	**95% CI**	***b***
Cohort						
2 (29 March to 7 April)	0.05*	0.01, 0.09	0.07	0.04	−0.00, 0.08	0.06
3 (8 to 18 April)	0.10***	0.06, 0.14	0.15	0.12***	0.07, 0.16	0.17
Outbreak-related media exposure (daily hours/week)	0.15***	0.10, 0.19	0.02	0.13***	0.08, 0.17	0.01
Relative media consumption	0.12***	0.08, 0.15	0.00	0.04*	0.00, 0.08	0.00
Conflicting info from news media	0.17***	0.13, 0.20	0.12	0.09***	0.05, 0.13	0.06
Personal exposures	0.09***	0.06, 0.13	0.15	0.11***	0.06, 0.15	0.17
Work exposures	−0.03	−0.06, 0.01	−0.04	−0.07***	−0.11, −0.03	−0.11
Community exposures	0.00	−0.04, 0.03	0.00	−0.01	−0.05, 0.02	−0.01
Secondary stressors	0.19***	0.15, 0.24	0.12	0.12***	0.07, 0.16	0.07
Prior mental health diagnoses	0.18***	0.13, 0.22	0.33	0.27***	0.22, 0.32	0.49
Prior physical health diagnoses	0.06**	0.02, 0.09	0.03	0.08***	0.04, 0.12	0.05
Age	−0.10***	−0.14, −0.06	−0.00	−0.18***	−0.23, −0.14	−0.01
Race/ethnicity						
Black, non-Hispanic	−0.01	−0.05, 0.03	−0.02	−0.04	−0.08, 0.00	−0.09
Other, non-Hispanic	−0.01	−0.04, 0.02	−0.02	−0.00	−0.03, 0.03	−0.01
Hispanic	0.01	−0.02, 0.05	0.03	0.03	−0.01, 0.07	0.07
Bachelor’s degree +	0.02	−0.01, 0.05	0.02	−0.03	−0.06, 0.01	−0.04
Female sex	0.12***	0.08, 0.15	0.17	0.02	−0.02, 0.05	0.02
Income	−0.02	−0.06, 0.02	−0.00	−0.08***	−0.12, −0.04	−0.03
Residential area						
Suburban	−0.03*	−0.07, −0.00	−0.08	−0.04**	−0.07, −0.01	−0.10
Town	0.01	−0.03, 0.04	0.01	−0.01	−0.04, 0.03	−0.02
Rural	0.01	−0.03, 0.05	0.03	0.00	−0.03, 0.04	0.01
Region						
Midwest	−0.07**	−0.12, −0.02	−0.11	−0.03	−0.08, 0.03	−0.04
South	−0.07**	−0.12, −0.02	−0.11	−0.03	−0.09, 0.03	−0.04
West	−0.06*	−0.11, −0.01	−0.09	−0.01	−0.07, 0.04	−0.02
Constant	0.00	−0.03, 0.03	1.23	0.02	−0.01, 0.05	0.60
Model statistics	*F*(24,6484.7) = 32.77; *P* < .001			*F*(24,6484.6) = 23.59; *P* < .001		
	*R^2^* = 0.272			*R^2^* = 0.244		

*P < 0.05.

**P < 0.01.

***P < 0.001.

We then examined personal, work-related, media-based, and secondary stress exposures to the COVID-19 outbreak as predictors of acute stress and depressive symptoms, after adjusting for demographics and pre–COVID-19 mental and physical health histories. Acute stress and depressive symptoms were associated with personal exposure to the outbreak (β = 0.09 and β = 0.11, respectively), but not community exposures (β = 0.00 and β = −0.01, respectively). Secondary stressors (e.g., job and wage loss) predicted higher acute stress (β = 0.19) and depressive symptoms (β = 0.12), and work-related exposures predicted lower depressive symptoms (β = −0.07).

Last, all three forms of media exposure predicted higher acute stress and depressive symptoms: hours of COVID-19–related media consumption (β = 0.15 and β = 0.13, respectively), increased media consumption relative to the participant’s pre-outbreak media behavior (β = 0.12 and β = 0.04, respectively), and higher frequency of exposure to conflicting information about the outbreak in the media (β = 0.17 and β = 0.09, respectively). Table S2 presents findings for each of the three individual cohorts. The pattern across all three cohorts was consistent with the findings reported above.

## DISCUSSION

We provide evidence that between 18 March and 18 April 2020, as the rates of COVID-19 positive cases and deaths increased substantially across the United States, COVID-19–related acute stress and depressive symptoms increased over time in the United States. These findings are consistent with studies linking the COVID-19 pandemic with declines in well-being around the globe (*5*, *24*, *25*). Unlike other studies, our unique study design allowed us to examine population-based trends in the early psychological consequences of the COVID-19 pandemic as it unfolded using a large, representative, probability-based national sample on whom prepandemic mental and physical health data were available (collected before the pandemic and hence not susceptible to concerns about recall bias). Three key findings in particular offer insights into ways to encourage community resilience when addressing a crisis of this magnitude: support individuals with preexisting conditions, mitigate secondary stress, and monitor extensive media exposure.

First, results indicate that individuals with preexisting mental and physical health diagnoses were more likely to exhibit both acute stress and depressive symptoms—having a history of prepandemic psychiatric diagnoses was the strongest predictor of depressive symptoms during the pandemic, highlighting the increased risk profile of individuals with preexisting conditions (*2*). These findings are consistent with those of other COVID-related studies including the probability-based Zurich Project on the Social Development from Childhood to Adulthood, a prospective longitudinal study of youth in Switzerland (*6*), and several nonprobability-based studies conducted in other countries (*5*, *7*). Prior life stress (e.g., bullying and other victimization) was also linked with young adults’ emotional responses to the pandemic (*6*). Together, these findings highlight the importance of prioritizing allocation of mental health services to individuals known to have prior victimization and/or mental health conditions.

Second, secondary stressors—job and/or wage loss and shortages of necessities—were strong predictors of both acute stress and depressive symptoms. Several previous studies have documented the negative mental health impact of secondary, ongoing stressors following different types of collective trauma (*26*, *27*), including the current COVID-19 pandemic (*6*). In the context of the COVID-19 pandemic, communities coping with combined effects of illness, death, job loss, and economic strain may benefit from early and efficient provision of support services to help prevent or mitigate the mental health risks associated with complex grief (*28*). By mitigating the impact of secondary stressors, such interventions could reduce the risk for experiencing a painful “loss spiral” in which stress begets psychological distress, which begets more stress (*29*). Addressing these potential threats to mental health would likely prove beneficial for physical health as well (*30*).

Third, consistent with recent COVID-19 studies, exposure to pandemic-related media coverage was associated with greater pandemic-specific acute stress and depressive symptoms (*2*, *14*). Daily hours of pandemic-related media exposure, increases in daily media use, and exposure to conflicting information in the news media all predicted acute stress and depressive symptoms. Frequency of exposure to conflicting information in news media was among the strongest predictors of pandemic-specific acute stress symptoms, suggesting the importance of providing consistent messaging to promote resilience and protect mental health when coping with an ambiguous collective stressor (*16*, *30*). As demonstrated after the 2014 Ebola public health outbreak in the United States, when given clear communication about risk and protective behaviors, the public can understand their contours and report risk assessments accurately (*31*). However, if conflicting media messages increase public perceptions of uncertainty about one’s own safety during the pandemic, they are likely to raise stress, anxiety, and depression levels (*32*), highlighting the potential for harm associated with poor risk communication conveyed in the media. Relying on social media sources for information during the pandemic may exacerbate this risk by increasing users’ negative affect, symptoms of stress, anxiety, and depression (*14*), and promoting conspiracy theories that undermine engagement in health behaviors (*21*). Given the degree to which the public relies on media sources for information during a crisis (*8*), it is critical for them to provide accurate information in a nonsensationalistic manner, using clear, noncontradictory messaging (*2*, *30*).

During the early weeks of the pandemic, media reports of growing numbers of infections and deaths, and the economic turmoil associated with sweeping public health interventions (e.g., closure of businesses and schools) to mitigate the escalating threat, undoubtedly raised anxiety. Akin to what we found when individuals reported distress associated with an approaching hurricane (*12*), increased media exposure to an impending threat is associated with distress and more media consumption over time, potentially creating a cycle of distress, especially if the threat, such as the pandemic, does not abate (*10*). Studies have further demonstrated that subjective reports of acute stress following collective and individual traumas are associated with risk perceptions (*33*), as well as subsequent physical health ailments, including higher risk of all-cause mortality (*34*). Acute stress has been associated with subsequent cardiovascular illness in large population-based studies (*35*), even when respondents’ exposure to collective stress (i.e., 9/11 attacks) was primarily through the media (*13*). Together, these findings suggest that heightened stress responses following media exposure may have important implications for the public’s physical health. Encouraging the public to limit their exposure to media is an important public health intervention to prevent mental and physical health symptoms and promote resilience (*2*).

In addition, personal exposure (e.g., self or close other tested positive to COVID-19) was associated with higher acute stress and depressive symptoms, whereas community-level exposures (e.g., stay-at-home orders) were not, suggesting that concerns about contracting the disease outweighed concerns about pandemic-related disruptions in daily life. Unlike big data findings suggesting that stay-at-home orders may “flatten the curve” of psychological symptoms (e.g., anxiety, hopelessness, and suicide) in the United States (*18*), our findings offer evidence that respondents’ acute stress and depressive symptoms continued to rise after the stay-at-home orders were implemented. Furthermore, our data suggest that individuals who continued to work during this early phase of the pandemic were less depressed than individuals who were not working, even though they were at greater risk for contracting the virus. It is possible that respondents who lost their jobs in the lockdown experienced a spike in depressive symptoms as unemployment is robustly linked with depression (*36*). Alternatively, remaining employed as an “essential” worker may have given new meaning to respondents’ work that reduced their risk for depression (*37*). Future research should address trends in specific types of exposures and their link to mental health outcomes over time as pandemic-related restrictions are relaxed.

In keeping with several recent studies (*19*, *25*, *38*), young individuals reported higher acute stress and depressive symptoms than older respondents, suggesting that despite being most deadly for older populations at the time of our data collection (*39*), the COVID-19 pandemic and its aftermath have had widespread impacts across populations. The heightened stress and depression among young people may reflect feelings of uncertainty about the future, or a foreshortened sense of the future (*40*), as efforts to control the pandemic have led to an economic downturn affecting future plans/expectations for millions of young people. How these age differences in the early mental health response to the pandemic affect the subsequent well-being of young people around the globe is another important topic for future research.

In this study, we provide three consecutive representative snapshots of early mental health responses weighted to a national sample to allow comparisons across cohorts over time. We acknowledge that without longitudinal data, we cannot address within-person change over time, and ongoing data collection will enable future examination of such change. Moreover, we acknowledge that a minority of individuals chose not to complete our survey during the fielding periods. Nonetheless, our sampling and weighting procedures ensure that we can make population estimates and draw conclusions accordingly.

We demonstrate that the COVID-19 pandemic and the media environment surrounding it are associated with higher acute stress and depressive symptoms in three consecutive, large cross-sectional representative samples of Americans. We used a nuanced approach to conceptualizing media exposure by assessing amount (from varied sources), content (conflicting information), and relative increase/decrease. The many potential downstream public health consequences of this unfolding, ambiguous pandemic stretch far beyond the number of cases and deaths directly due to the novel coronavirus itself. Future research should address the long-term public health impacts of the multiple threats of preexisting risk, ongoing, secondary stressors, and media-related psychological distress. This information is critical for promoting resilience through effective communication and early interventions targeting public health and well-being during this unprecedented health crisis.

## MATERIALS AND METHODS

### Data collection and sample

The survey was conducted using NORC’s AmeriSpeak panel, a probability-based panel of 35,000 U.S. households. AmeriSpeak panel households are selected at random from across the United States to form a representative cross section of U.S. households. NORC’s AmeriSpeak panel is the only probability panel in the United States that uses random door-to-door interviewing to recruit its participants, who subsequently participate in AmeriSpeak surveys by web or telephone. As a result, AmeriSpeak attains response rates nearly three times higher than other probability panels in the United States (*41*). Unlike typical internet panels, for which people who already have internet access choose to opt in, no one can volunteer for the AmeriSpeak panel.

NORC drew our stratified random sample of 11,139 panelists from the AmeriSpeak panel using sample stratification to assure representativeness with respect to age, gender, race/ethnicity, and education. NORC fielded a 20-min survey for 10 days each to three consecutive cohorts of 3713 panelists (cohort 1, 18 to 28 March 2020; cohort 2, 29 March to 7 April 2020; cohort 3, 8 to 18 April 2020); participants received notice that the survey was available via a password-protected email address and completed the survey online anonymously. Surveys were confidential, self-administered, and accessible any time for the designated period; participants could complete a survey only once. Respondents received a small compensation (cash equivalent US$4) for completing the survey. When the fielding period ended, 6598 had completed surveys (59.2% completion rate); 84 cases (1.3%) were removed from the final sample owing to unreliable survey completion times (under 6.5 min) or extensive missing data (>50% of questions), leaving *N* = 6514 (*n* = 2122, *n* = 2234, and *n* = 2158 respondents for cohorts 1, 2, and 3, respectively) in the final sample for analysis. Using standard definitions for survey response rate reporting proposed by the American Association for Public Opinion Research (*42*), the survey cooperation rate was 58.5%.

Across all cohorts, ~85% of respondents completed the survey within the first 3 days of its fielding; surveys were completed on computers (44%), smartphones (54%), and tablets (2%). Before 1 January 2020, and thus before the start of the COVID-19 outbreak in the United States, all respondents had completed mental and physical health assessments; we examined pandemic-related acute stress and depressive symptoms, controlling for these baseline data. Participants provided informed consent when they joined the NORC panel and were informed that their identities would remain confidential. All research activities were reviewed and approved by the University of California, Irvine Institutional Review Board for Human Subjects research.

### Measures

Participants’ demographics (including age, race/ethnicity, education, gender, income, geographic region of residence, and residential area such as urban or rural) and health information were collected by NORC upon enrollment into the AmeriSpeak panel and updated periodically for accuracy; 56% of the sample completed pre-COVID health data in 2019, 25% completed it in 2018, and 19% completed it in 2017. Participants reported whether a doctor had ever diagnosed them with several physical and mental health ailments. Prior mental health diagnoses were coded as 0 (no prior mental health diagnosis) or 1 (prior anxiety, depression, or any other emotional, nervous, or psychiatric diagnosis). Prior physical health diagnoses were coded as a count of eight possible prior diagnoses (i.e., high cholesterol, hypertension, diabetes/high blood sugar, heart disease, stroke, cancer, lung disease, and other diagnoses). Acute stress responses to the COVID-19 outbreak were assessed using a modified version of the Acute Stress Disorder Scale 5 (*43*). Participants used a five-point scale (1, “not at all”; 5, “a great deal”) to report the degree to which they had experienced 10 symptoms of acute stress as a result of the COVID-19 outbreak in the previous week (α = 0.86). Depressive symptoms were assessed with the depression subscale of the Brief Symptom Inventory-18 (*44*). Participants used a five-point scale (0, “not at all”; 4, “extremely”) to report the degree to which they experienced six items in the past week (α = 0.86).

Participants completed a checklist to report their degree of exposure to the COVID-19 outbreak. Ten items reflected personal exposures: direct or indirect disease exposure (e.g., I/someone close to me was diagnosed with coronavirus); two items reflected work exposures (e.g., my job requires in-person interaction and I am still working); and six items reflected community exposures: community-wide outbreak-related impacts (e.g., my community has been instructed to “shelter in place”). Seven items reflected COVID-19–related secondary stressors (e.g., lost job and canceled travel plans). Four scores comprised counts of each of these personal, work, and community exposures, and secondary stressors; because of high skewness in the personal exposures subscale, responses to these items were dichotomized for analyses.

Media exposure to the COVID-19 outbreak was assessed using participants’ reports of the number of hours per day (0 to 11+) spent in the previous week engaging with each of three sources of media coverage of the outbreak: traditional media (i.e., TV, radio, and print news), online news, and social media (e.g., Facebook, Reddit, and Twitter). The COVID-19–related media coverage score reflected a sum of total daily hours of media exposure across these three sources. Because participants could simultaneously engage with multiple sources, the maximum score was 33. Participants then used a sliding scale to report how much more or less they were engaging with news media than they were before the coronavirus outbreak; positive responses indicated an increase from their pre-outbreak behavior, and negative responses indicated a decrease (possible range: −100 to 100; 0 = about the same). Participants also reported how often they felt they were receiving “conflicting or confusing information” from the news media over the previous week using a five-point scale (1, “never”; 5, “all the time”).

### Analytic strategy

Statistical analyses were conducted using Stata 16.1 (StataCorp, College Station, TX). All data were weighted to adjust for probability of selection into the AmeriSpeak panel and to account for differences between our sample and U.S. Census benchmarks (*23*). Poststratification weights were iteratively constructed from respondents’ design weights using probability estimates based on age, gender, race/ethnicity, education, and census region. The weighted sample closely matches the February 2020 U.S. Census data (see table S1) (*23*). Mean scores for acute stress and depressive symptoms were computed to capture variability in response (*45*). We constructed multiple ordinary least squares (OLS) regression models to examine predictors of the acute stress in response to the COVID-19 outbreak and depressive symptoms. To account for missing data, the model was estimated using a multiple imputation using chained equations method. This method generates multiple possible observations for each missing value to create a pooled set of final estimates and robust standard errors for the model that accounts for uncertainty in variables with missing data. Because of low missingness across variables (0.02 to 2.76% missingness for individual variables), a total of 20 imputations was used. Acute stress and depressive symptoms were regressed on demographics; cohort membership; pre-outbreak mental and physical health ailments; personal, work, and community exposure to the outbreak; secondary stressors; hours of COVID-19–related media coverage consumed; relative media consumption compared to pre-outbreak levels; and the degree to which participants were exposed to conflicting or confusing information via the media.

## Supplementary Material

abd5390_SM.pdf

## SUPPLEMENTARY MATERIALS

Supplementary material for this article is available at http://advances.sciencemag.org/cgi/content/full/sciadv.abd5390/DC1
