# Supplementary material for: The unfolding COVID-19 pandemic: A probability-based, nationally representative study of mental health in the United States
Source: Sci Adv. 2020 Oct 14;6(42):eabd5390. doi: 10.1126/sciadv.abd5390 (PMC7556755; doi:10.1126/sciadv.abd5390)
Supplement: abd5390_SM.pdf [file abd5390_SM.pdf]

Science Advances
AAAS

advances.sciencemag.org/cgi/content/full/sciadv.abd5390/DC1

Supplementary Materials for

The unfolding COVID-19 pandemic: A probability-based, nationally representative study of mental health in the U.S.

E. Alison Holman*, Rebecca R. Thompson, Dana Rose Garfin and Roxane Cohen Silver*

*Corresponding authors. Email: aholman@uci.edu, rsilver@uci.edu

Published 18 September 2020, *Sci. Adv.* **6**, eabd5390 (2020)
DOI: 10.1126/sciadv.abd5390

This PDF file includes:

Tables S1 to S4

Supplemental Table 1. Weighted UCI Sample Demographics and March 2019 Current Population Survey Benchmarks

	Weighted UCI Sample	Current Population Survey Benchmarks
**Age**	**weighted percent**	**benchmark percent**
1. 18 - 24	10.9483	11.6077
2. 25 - 29	9.3293	9.2897
3. 30 - 39	17.4486	17.3109
4. 40 - 49	16.09	15.9356
5. 50 - 59	16.6576	16.5699
6. 60 - 64	8.2981	8.2184
7. 65 +	21.2282	21.0677
**Gender**		
1. Male	48.0561	48.4112
2. Female	51.9439	51.5888
**Region**		
1. New England	4.7222	4.6769
2. Middle Atlantic	12.5841	12.7861
3. East North Central	14.4424	14.3038
4. West North Central	6.5326	6.4699
5. South Atlantic	19.8536	20.2663
6. East South Central	5.889	5.8324
7. West South Central	11.9158	11.835
8. Mountain	7.5494	7.477
9. Pacific	16.511	16.3526
**Education**		
1. < High School	9.8567	10.6002
2. High School	28.4668	28.3151
3. > High School < college	28.0376	27.7685
4. >= college	33.6389	33.3161
**Race/Ethnicity**		
1. Non-Hispanic White	63.5886	63.1365
2. Non-Hispanic Black	11.7533	11.8207
3. Hispanic	15.9819	16.4499
4. All Other	8.6761	8.5929

Supplemental Table 2. Descriptive statistics for model covariates by cohort (*N*=6,514).

Variable	Cohort 1	Cohort 2	Cohort 3
Age, *M*(SD)	47.65 (17.50)	47.53 (17.67)	47.67 (17.36)
Sex, N (weighted %)			
Male	875 (48.41%)	849 (48.41%)	849 (48.41%)
Female	1,247 (51.59%)	1,385 (51.59%)	1,385 (51.59%)
Race/Ethnicity, N (weighted %)			
White, non-Hispanic	1,606 (63.14%)	1,699 (63.14%)	1,624 (63.14%)
Black, non-Hispanic	175 (11.82%)	155 (11.82%)	184 (11.82%)
Other/2+ races, non-Hispanic	160 (8.59%)	187 (8.59%)	174 (8.59%)
Hispanic	181 (16.45%)	193 (16.45%)	176 (16.45%)
Education, N (weighted %)			
Less than Bachelor’s degree	1,162 (66.68%)	1,206 (66.68%)	1,201 (66.68%)
Bachelor’s degree +	960 (33.32%)	1,028 (33.32%)	957 (33.32%)
Income, Median	$40,000-$49,999	$40,000-$49,999	$40,000-$49,999
Residential Area, N (weighted %)			
Urban	1,384 (64.21%)	1,542 (68.35%)	1,445 (66.75%)
Suburban	220 (11.22%)	197 (9.12%)	201 (11.04%)
Town	279 (13.41%)	268 (11.90%)	281 (12.77%)
Rural	239 (11.17%)	227 (10.63%)	231 (9.44%)
Region, N (weighted %)			
Northeast	330 (17.46%)	343 (17.46%)	326 (17.46%)
Midwest	559 (20.77%)	564 (20.77%)	567 (20.77%)
South	673 (37.93%)	707 (37.93%)	655 (37.93%)
West	560 (23.83%)	620 (23.83%)	610 (23.83%)
Prior mental health diagnoses, N (weighted %)			
Yes	1,747 (83.11%)	1,861 (83.80%)	1,749 (79.89%)

No	375 (16.89%)	373 (16.20%)	408 (20.11%)
Prior physical health diagnoses, *M*(SD)	1.08 (1.26)	1.00 (1.21)	1.07 (1.22)
Outbreak-related media exposure, *M*(SD)	7.41 (6.91)	7.38 (7.17)	6.46 (6.44)
Relative media consumption, *M*(SD)	28.79 (44.35)	27.29 (47.98)	21.98 (49.47)
Conflicting info from news media, *M*(SD)	2.91 (1.02)	2.94 (1.04)	3.02 (1.07)
Personal exposures, N (weighted %)			
Yes	1,776 (83.16%)	1,683 (75.71%)	1,505 (70.04%)
No	342 (16.73%)	549 (24.29%)	647 (29.96%)
Work exposures, N (weighted %)			
Yes	1,463 (67.71%)	1,632 (71.09%)	1,561 (71.39%)
No	644 (31.80%)	594 (28.91%)	583 (27.19%)
Community exposures, *M*(SD)	4.60 (1.42)	4.97 (1.55)	5.15 (1.43)
Secondary stressors, *M*(SD)	1.25 (1.19)	1.40 (1.21)	1.48 (1.23)

Note: All means, standard deviations, and proportions are based on weighted data; *n*s are unweighted.

Supplemental Table 3. Regression coefficients for models predicting pandemic-related acute stress across the three cohorts

	Cohort 1 (*n*=2122)			Cohort 2 (*n*=2234)			Cohort 3 (*n*=2158)		
**Predictor Variables**	**β**	**95% CI**	***b***	**β**	**95% CI**	***b***	**β**	**95% CI**	***b***
Outbreak-related media exposure (daily hours/week)	0.15***	0.07, 0.22	0.02	0.14***	0.07, 0.22	0.02	0.16***	0.07, 0.24	0.02
Relative media consumption	0.16***	0.09, 0.22	0.00	0.04	-0.02, 0.11	0.00	0.15***	0.08, 0.22	0.00
Conflicting info from news media	0.16***	0.09, 0.22	0.10	0.19***	0.13, 0.25	0.13	0.14***	0.08, 0.20	0.10
Personal exposure	0.10**	0.04, 0.17	0.19	0.11**	0.05, 0.17	0.18	0.07*	0.01, 0.13	0.11
Work exposure	-0.08**	-0.14, -0.02	-0.12	-0.01	-0.07, 0.06	-0.01	0.01	-0.05, 0.08	0.02
Community exposures	0.02	-0.03, 0.07	0.01	0.00	-0.06, 0.07	0.00	-0.02	-0.08, 0.04	-0.01
Secondary stressors	0.16***	0.07, 0.25	0.10	0.21***	0.14, 0.28	0.12	0.18***	0.11, 0.26	0.11
Prior mental health Dx	0.11***	0.05, 0.17	0.20	0.19***	0.10, 0.27	0.35	0.23***	0.15, 0.30	0.43
Prior physical health Dx	0.06	-0.00, 0.12	0.03	0.02	-0.04, 0.09	0.01	0.09**	0.03, 0.16	0.06
Age	-0.13***	-0.19, -0.06	-0.01	-0.04	-0.11, 0.02	-0.00	-0.15***	-0.22, -0.07	-0.01
Race/Ethnicity									
Black, Non-Hispanic	-0.08**	-0.14, -0.03	-0.20	-0.01	-0.08, 0.06	-0.02	0.02	-0.04, 0.08	0.05
Other, Non-Hispanic	0.01	-0.04, 0.05	0.02	-0.02	-0.08, 0.03	-0.06	-0.00	-0.06, 0.05	-0.01
Hispanic	0.03	-0.03, 0.10	0.09	0.02	-0.04, 0.08	0.04	-0.01	-0.06, 0.05	-0.02
Bachelor's degree +	0.06*	0.01, 0.11	0.07	0.05	-0.00, 0.10	0.06	-0.03	-0.08, 0.03	-0.04
Female gender	0.12***	0.07, 0.18	0.18	0.09**	0.03, 0.15	0.12	0.15***	0.09, 0.21	0.23
Income	-0.05	-0.12, 0.01	-0.01	-0.00	-0.08, 0.07	-0.00	0.01	-0.06, 0.07	0.00
Residential area									
Suburban	-0.03	-0.07, 0.02	-0.06	-0.06	-0.11, 0.00	-0.13	-0.04	-0.10, 0.02	-0.10

Town	-0.02	-0.07, 0.03	-0.04	0.04	-0.02, 0.09	0.08	-0.02	-0.07, 0.03	-0.04
Rural	0.02	-0.03, 0.08	0.05	0.01	-0.05, 0.07	0.03	-0.01	-0.06, 0.04	-0.02
Region									
Midwest	-0.08	-0.17, 0.01	-0.12	-0.02	-0.09, 0.04	-0.04	-0.10*	-0.20, -0.01	-0.17
South	-0.15**	-0.24, -0.06	-0.22	0.03	-0.05, 0.10	0.04	-0.10	-0.20, 0.00	-0.16
West	-0.11*	-0.20, -0.02	-0.17	0.02	-0.05, 0.09	0.03	-0.10*	-0.20, -0.00	-0.16
Constant	-0.02	-0.07, 0.02	1.39	-0.02	-0.06, 0.02	1.05	0.02	-0.03, 0.07	1.52
Model Statistics	$F(22, 2095.9) = 11.62$; $p<.001$			$F(22, 2208.9) = 13.19$; $p<.001$			$F(22, 2132.3) = 16.41$; $p<.001$		
	$R^2 = 0.298$			$R^2 = 0.245$			$R^2 = 0.314$		

Note: reference group for ethnicity is white, non-Hispanic; reference group for residential area is urban; reference group for region is Northeast. All models were estimated using sampling weights to account for sampling design and differences between the sample and U.S. census benchmarks.

Supplemental Table 4. Regression coefficients for models predicting depression across the three cohorts

	Cohort 1 (*n*=2122)			Cohort 2 (*n*=2234)			Cohort 3 (*n*=2158)		
**Predictor Variables**	**β**	**95% CI**	***b***	**β**	**95% CI**	***b***	**β**	**95% CI**	***b***
Outbreak-related media exposure (daily hours/week)	0.10**	0.03, 0.17	0.01	0.13**	0.05, 0.20	0.01	0.16**	0.06, 0.25	0.02
Relative media consumption	0.03	-0.04, 0.11	0.00	0.02	-0.04, 0.08	0.00	0.06	-0.01, 0.13	0.00
Conflicting info from news media	0.09*	0.02, 0.16	0.05	0.10**	0.04, 0.16	0.06	0.09**	0.02, 0.15	0.06
Personal exposure	0.08*	0.01, 0.15	0.14	0.10**	0.04, 0.17	0.15	0.13***	0.06, 0.20	0.21
Work exposure	-0.12***	-0.19, -0.06	-0.18	-0.03	-0.09, 0.04	-0.04	-0.06	-0.14, 0.02	-0.10
Community exposures	-0.01	-0.07, 0.05	-0.01	-0.03	-0.09, 0.04	-0.01	0.03	-0.04, 0.10	0.02
Secondary stressors	0.10	-0.01, 0.22	0.06	0.16***	0.09, 0.22	0.09	0.06	-0.02, 0.15	0.04
Prior mental health Dx	0.22***	0.14, 0.30	0.38	0.27***	0.17, 0.36	0.48	0.30***	0.21, 0.39	0.56
Prior physical health Dx	0.10**	0.03, 0.17	0.05	0.05	-0.02, 0.12	0.03	0.09*	0.02, 0.16	0.05
Age	-0.18***	-0.27, -0.10	-0.01	-0.13***	-0.19, -0.07	-0.01	-0.26***	-0.36, -0.16	-0.01
Race/Ethnicity									
Black, Non-Hispanic	-0.08*	-0.14, -0.02	-0.20	-0.06	-0.13, 0.02	-0.14	-0.01	-0.07, 0.05	-0.03
Other, Non-Hispanic	-0.03	-0.08, 0.02	-0.07	0.02	-0.03, 0.08	0.06	-0.01	-0.07, 0.05	-0.03
Hispanic	0.05	-0.02, 0.12	0.12	0.03	-0.03, 0.08	0.06	0.01	-0.07, 0.08	0.02
Bachelor’s degree +	-0.00	-0.06, 0.06	-0.01	0.03	-0.02, 0.09	0.04	-0.10**	-0.15, -0.04	-0.14
Female gender	0.00	-0.06, 0.07	0.01	-0.02	-0.08, 0.05	-0.02	0.06	-0.01, 0.12	0.09
Income	-0.06	-0.14, 0.02	-0.02	-0.12**	-0.19, -0.05	-0.04	-0.05	-0.13, 0.02	-0.02
Residential area									
Suburban	-0.07**	-0.11, -0.03	-0.15	-0.05*	-0.10, -0.01	-0.13	-0.02	-0.08, 0.04	-0.05

Town	-0.02	-0.09, 0.05	-0.03	0.01	-0.05, 0.07	0.01	-0.03	-0.08, 0.02	-0.07
Rural	0.00	-0.06, 0.06	0.01	-0.02	-0.07, 0.04	-0.03	0.02	-0.05, 0.08	0.04
Region									
Midwest	-0.03	-0.12, 0.06	-0.04	0.03	-0.04, 0.10	0.05	-0.07	-0.17, 0.04	-0.12
South	-0.05	-0.16, 0.05	-0.07	0.06	-0.01, 0.14	0.09	-0.08	-0.18, 0.03	-0.13
West	-0.04	-0.13, 0.06	-0.05	0.08*	0.00, 0.15	0.12	-0.06	-0.16, 0.04	-0.10
Constant	0.00	-0.05, 0.06	0.66	0.00	-0.04, 0.05	0.49	0.04	-0.01, 0.09	0.84
Model Statistics	$F(22, 2096.3) = 7.25$; $p<.001$			$F(22, 2208.9) = 11.48$; $p<.001$			$F(22, 2132.4) = 11.59$; $p<.001$		
	$R^2 = 0.199$			$R^2 = 0.239$			$R^2 = 0.297$		

Note: reference group for ethnicity is white, non-Hispanic; reference group for residential area is urban; reference group for region is Northeast. All models were estimated using sampling weights to account for sampling design and differences between the sample and U.S. census benchmarks.
